# *Moringa oleifera*-based feed supplement protects against renal ischaemia/reperfusion injury via downregulation of Bax/caspase 3 signaling

**DOI:** 10.3389/fnut.2024.1396864

**Published:** 2024-04-23

**Authors:** Oladele A. Afolabi, Tunmise M. Akhigbe, Sodiq. O. Hammed, Moses A. Hamed, Victor O. Ekundina, Richard A. Ajike, Babatunde A. Alabi, Roland E. Akhigbe

**Affiliations:** ^1^Department of Physiology, Ladoke Akintola University of Technology, Ogbomoso, Oyo, Nigeria; ^2^Department of Agronomy, Osun State University, Osogbo, Nigeria; ^3^Reproductive Biology and Toxicology Research Laboratory, Oasis of Grace Hospital, Osogbo, Nigeria; ^4^The Brainwill Laboratory, Osogbo, Oyo, Nigeria; ^5^Department of Medical Laboratory Science, Afe Babalola University, Ado Ekiti, Ekiti, Nigeria; ^6^Department of Pharmacology, Bowen University, Iwo, Nigeria

**Keywords:** apoptosis, caspase, inflammation, intestinal torsion, oxidative stress, supplement

## Abstract

**Introduction:**

Ischaemia/reperfusion (I/R) may lead to acute kidney injury via the induction of oxidative stress. On the other hand, Moringa oleifera has been reported to exert antioxidant activities. This study was designed to assess whether or not Moringa oleifera-based feed supplement could prevent I/R-induced renal injury.

**Materials and methods:**

Renal I/R was induced by occluding the right renal artery for 30  min followed by a 2-h reperfusion.

**Results:**

Renal I/R led to increased absolute renal weight and renal organo-somatic weight index. Renal I/R also caused distortion of renal histoarchitecture and impaired renal function evidenced by elevated serum creatinine and blood urea nitrogen. In addition, renal I/R significantly elevated renal levels of hydrogen peroxide, MDA, and advanced oxidation protein products, but suppressed the levels of reduced glutathione, protein thiol, and non-protein thiol, and the activities of superoxide dismutase and glutathione peroxidase. In addition, renal I/R up-regulated myeloperoxidase activity and the renal levels of NO, TNF-α, and IL-6. Renal I/R also up-regulated Bax and caspase 3 expression in the kidney. Furthermore, I/R-driven structural and biochemical alterations were markedly inhibited by Moringa oleifera-based feed supplement.

**Discussion:**

These results suggest that Moringa oleifera-based feed supplement may preserve the gross and histoarchitectural integrity of the kidney as well as renal function via downregulation of Bax/caspase 3 signaling by targeting oxidative stress, inflammation and apoptosis in the kidney of I/R rat.

## Introduction

The kidney is a major homeostatic organ of the body which regulates water balance, erythropoiesis blood pressure, electrolyte balance and numerous other systems of the body. One of the major functions of the kidney is to filter and excrete nitrogenous waste products from the circulation, and to re-absorb water and essential electrolytes into the circulation. Hence, raised blood urea nitrogen or serum urea, and serum creatinine are markers of impaired renal function (1–3), which are usually seen in acute kidney injury (AKI). Acute kidney injury is a complication of renal ischemia–reperfusion injury seen in patients after post-surgical procedures in the kidney such as transplant, delayed graft function and graft rejection (4). Consequent upon its widespread homeostatic function, renal ischemia/reperfusion (I/R) injury, IRI, is a urological emergency and treatment must be urgent to prevent multiple organ damage in the long run.

The mechanisms through which IRI causes damage to the kidney include release of reactive oxygen species (ROS), activation of neutrophils and inflammatory mediators (cytokines), and upregulation of other inflammatory mediators like adhesion molecules (5). Several studies have shown that the aetiology of renal damage starts from the ischaemic phase. During the ischaemic phase, there is accumulation of ROS such as hydrogen peroxide, superoxide radicals and hydroxyl radicals due to obstruction of blood flow to the kidney (6). These radicals, in severe ischaemic condition, interact with other radicals to form reactive nitrogen species (RNS) and peroxynitrite radicals, which interfere with cellular structures such as lipids, proteins, and DNA to aggravate renal damage (7). On the other hand, reperfusion of the ischaemic renal tissue meant to restore blood to the already starved tissue further increases free radical production and triggers inflammatory responses, which worsens tissue damage (8). Therefore, effective treatment approach that would reduce tissue damage must be targeted at not only reperfusion injury but also ischemic injury; after all, inflammation, renal cell death and acute kidney failure are possible outcomes of reperfusion injury on the kidney, whose foundations are laid during the ischemic phase (5).

Several reports available in the literature have proven that medicinal herbs with antioxidant properties may be utilized in the management IRI (9). *Moringa oleifera* (MO) is one of such herbs reported in literature to exhibit antioxidant and free radical scavenging properties (10). MO, also known as drumstick tree, belongs to the family *Moringaceae* (11). It has been shown to possess analgesic, antipyretic, anti-diabetic, and hypotensive properties (10, 12) as well as antimicrobial/antibacterial (13), anti-inflammatory (14, 15), and antioxidant (11, 16) activities. Studies have also demonstrated the renoprotective potential of MO (17–20). Studies have ascribed the medicinal activities of MO to its phytochemical constituents such as carotenoids, vitamins, minerals, amino acids, saponins, terpenoid, sterols, glycosides, alkaloids, flavonoids, tannins, anthraquinones, and phenolics (11, 12, 21, 22). Although most experimental studies have reported the medicinal values of MO extracts, there are compelling pieces of evidence that demonstrated that MO-based food supplements, as commonly used in Nigeria, have nutritional and medicinal value (23, 24). Most experimental studies on herbal remedies ability to reduce the adverse effects of IR have involved pretreatment of animals for variable periods of time preceding the actual induction of IRI (25, 26). Although the experiments have by and large yielded promising results, such results could not be translated to real life situations due to the unpredictable nature of the occurrence of IRI. Therefore real-time efforts to alleviate the adverse effects of IRI must be directed at employing methods that can be applied if and when IRI occurs. The incorporation of MO into the diet to prevent nutritional deficiencies such as anemia and protein deficiency have been well documented (27, 28). Given the well documented antioxidant, anti-apoptotic and anti-inflammatory properties of MO, it is possible that incorporating it in the regular diet could greatly reduce the adverse effects of IRI.

Although ROS-induced apoptosis has been shown to play a role in I/R injury (29, 30), there is a dearth of data reporting the role of Bcl-2-associated X protein (Bax) and cysteine-aspartic protease 3 (caspase 3) in renal I/R injury. Caspase 3 may be activated through a death receptor (FAS, FAS-L), the endoplasmic reticulum, or the mitochondrial pathway (related to Bax) (31). Upregulation of Bax triggers caspase 3 activation, which in turn induces apoptosis (32, 33). Also, there is no report on the impact of MO-based food supplement, as popular used in some parts of Nigeria, on I/R-induced renal injury. Keeping in view above details, an effort was made to investigate whether or not Bax/caspase 3 signaling plays a role in I/R-induced renal injury. Also, we evaluated whether MO-based food supplement could confer renoprotection by inhibiting Bax/caspase 3 pathway apart from its beneficial antioxidant and anti-inflammatory activities. This study provides new evidence that supports the use of MO-based food supplement as a prophylactic measure in the prevention of intestinal I/R-induced renal injury.

## Materials and methods

### Experimental animals

Twenty-four (24) 12-week old male Wistar rats, weighing about 180–200 g, were obtained from the animal house of the department. They were acclimatized for 2 weeks, kept in well aerated plastic cages under natural conditions and fed with rat feeds and water *ad libitum.*

### Ethical approval

Ethical approval was issued by The Ministry of Health, Oyo State, Nigeria (Approval number: AD13/479/44406).

### Plant collection

Fresh leaves of *Moringa oleifera* were harvested from the university farm of Ladoke Akintola University of Technology (LAUTECH), Ogbomoso, Oyo State Nigeria. The leaves were authenticated by Dr. Mrs. Ogundola of Botany Unit, Department of Pure and Applied Biology, LAUTECH. The name of the plant was confirmed on http://www.theplantlist.org (accessed on 20 July 2021). A voucher specimen, LHO 616, was obtained and kept in the Herbarium at the Department of Botany, LAUTECH, Nigeria.

### *Moringa oleifera-*based feed supplement formulation

Low-dose and high-dose MO-based feed supplements were made by fortifying the normal rat diet with 10 and 20% of dried MO powder, respectively, as earlier described by Abioye and Aka (34). Low-dose MO-based supplement (10%) was formulated by mixing 22.5 kg of normal rat diet with 2.5 kg of MO powder, while high-dose MO-based supplement (20%) was formulated by mixing 20 kg of normal rat diet with 5 kg of MO powder. They were both mixed thoroughly to attain homogeneity and pelletized at the pelletizing unit of the Animal House of the department.

### Experimental design

After 2 weeks of acclimatization, the rats were randomly assigned to four groups (*n* = 6); the sham-operated, renal ischaemia-reperfusion (I/R), 1/R with 10% MO diet, and I/R with 20% MO diet. Animal in the I/R group underwent renal I/R procedure, while those in 1/R with 10% MO diet and I/R with 20% MO diet had 10% MO fortified diet and 20% MO fortified diet, respectively, for 14 days before the I/R procedure. The sham-operated and I/R rats were on the normal rat chow.

### Procedure for inducing renal ischaemia-reperfusion injury

Animals were prepared for surgical induction as earlier stated (35, 36). The rats were weighed and anesthetized with 10 mg/kg of Xylazine and 50 mg/kg of Ketamine. The abdomen was cleaned with 10% povidone iodine, a ventral midline incision was made on the abdomen and the right kidney was located. The right renal artery was spotted and clamped with a non-crushing forceps to induce renal ischaemia and sutured with 2–0 chromic suture. After 30 min of ischaemia, the rats were opened up and the clamp was removed to induce renal reperfusion for 2 h. After the specified period of reperfusion, the rats were sacrificed for blood and kidney collection. Asepsis was ensured and anesthesia maintained throughout the surgical procedure.

### Sacrifice and organ collection

Animals were euthanized under ketamine/xylazine. Blood samples were collected through the retro-orbital plexus. The right kidney of each animal was harvested, cleared of adherent tissues and weighed. The harvested kidney was cut longitudinally such that, one section was submerged inside formaldehyde for histological assessment while the other section was homogenized for the assessment of biochemical parameters.

### Kidney homogenate preparation

A regular weight of 0.5 g of the right renal mass was measured from the sacrificed animals and placed inside a universal bottle containing 2.5 mL of cold phosphate buffer (pH = 7.2). The samples were homogenized using a glass homogenizer. The homogenates were centrifuged at 2,500 revolutions per minutes for 15 min using cold centrifuge. The obtained supernatant was collected into an Eppendorf bottle and refrigerated properly for further analysis. Two microliters 2 μL of 2-vinylpyridine was added to the homogenate to prevent oxidation of GSH (37).

### Body weight and kidney weights

The body and renal weights of the animals were recorded using electronic weighing scale (Lisay, China). The renal organo-somatic index (OSI) was determined as renal weight/body weight X 100% (38).

### Estimation of biochemical parameters

Serum urea and creatinine were assayed using enzymatic colorimetric method (Randox Laboratory Ltd., Antrim, UK). Blood urea nitogen, BUN, was determined as urea/2.1428 (3).

Oxidative stress markers and antioxidants in the renal tissue were assayed using established methods as earlier reported; hydrogen peroxide concentration (39), malondialdehyde, MDA, levels (40), reduced glutathione, GSH, content (41), superoxide oxidase, SOD, activity (42), glutathione peroxidase, GPx, activity (43), protein thiol and non-protein thiol (44, 45), and advanced oxidation protein products, AOPP, levels (46).

The method of hydrogen peroxide assay employs a color reagent that contains xylenol orange dye in an acidic solution with sorbitol and ferrous ammonium sulfate which reacts to give up a purple color. The assay mixture was thoroughly mixed until it foamed. After incubation at room temperature for 30 min, a pale pink color complex was generated, and the absorbance was read against blank (distilled water) at 560 nm wavelength.

This method for determining MDA was based on the reaction between 2-thiobarbituric acid (TBA) and malondialdehyde, an end product of lipid peroxidation. On heating in acidic pH, a pink chromogen complex ([TBS] 2-malondialdehyde adduct) is formed and measured by its absorbance at 532 nm.

For the determination of GSH, an aliquot of the sample was deproteinized by the adding equal volume of 4% sulfosalicylic acid, which was centrifuged for 5 min at 4,000 rpm, then 0.5 mL of the supernatant was added to 4.5 mL of Ellman’s reagent. A blank was prepared with 0.5 mL of the diluted precipitating agent and 4.5 mL of Ellman’s reagent. Reduced glutathione, GSH, is equal to the absorbance at 412 nm.

For the assay of SOD, 1 mL of the sample was diluted in 9 mL of distilled water to make a 1 in 10 dilutions. An aliquot of 0.2 mL of the diluted sample was added to 2.5 mL of 0.05 M carbonate buffer (pH 10.2) and the reaction initiated by adding 0.3 mL of freshly prepared 0.3 mM adrenaline to the mixture that was rapidly mixed by inversion. The increase in absorbance was monitored every 30 s for 150 s 480 nm.

For GPx assay, the reaction mixture containing the sample was incubated for 3 min at 37°C, then 0.5 mL of 10% trichloroacetic acid (TCA) was added and centrifuged for 5 min at 3000 rpm. To 1 mL of each of the supernatants, 2 mL of phosphate buffer and 1 mL of 5′-5′- dithiobis-(2-dinitrobenzoic acid) (DTNB) solution was added, and the absorbance was read at 412 nm against a blank.

Renal nitric oxide (NO) content (47) and myeloperoxidase activity (48) were determined by colorimetry using established methods. Renal TNF-α, and IL-6 were determined using ELISA kits (Elabscience Biotechnology Co., Ltd., USA) per manufacturer’s guidelines.

### Histopathological examination and immunohistochemistry

Histopathological evaluation was performed as previously reported (3, 38). The renal tissues were transversely cut in slabs of about 0.5 cm thick and fixed in Bouin’s fluid for 24 h and then transferred to 70% alcohol for dehydration. The tissues were passed through 90% alcohol and chloroform for different durations before they were transferred into two changes of molten paraffin wax for 20 min each in an oven at 57°C. Serial sections were cut using rotary microtome at 5 microns. Slides were prepared from these tissues. The slides were de-waxed for about 15 min and passed through absolute alcohol, 95 and 70% alcohol and then rinsed with water for 5 min. The slides were then stained with Hematoxylin and Eosin. Photomicrographs were taken at X 100 and X 400 magnifications.

Formalin-fixed and paraffin-embedded renal tissues were sectioned at about 4 μm for immunohistochemistry for the assessment of Bax protein and caspase 3 expressions as earlier reported (49).

### Statistical analysis

Data were analyzed using Graph pad prism 7.0 (GraphPad Software Inc., La Jola, CA, USA) and presented as mean ± SD. One-way analysis of variance (ANOVA) followed by Tukey’s post-hoc test was conducted for multiple comparisons. A two-tailed *p*-value <0.05 was considered statistically significant.

## Results

The active bioactive components of MO, based on gas chromatography mass spectrophotometric (GC–MS) analysis, was reported in our previous study (36) as thiosemicarbazone, hydrazine, 1,3-dioxolane, octanoic acid, 1,3-benzenediamine, 9-octadecenoic acid, oleic acid, nonadecanoic acid, 3-undecanone, phosphonic acid, and cyclopentanecarboxylic acid.

### Effect of MO-based feed supplement on renal weight and renal OSI in I/R rats

**Figure 1:**
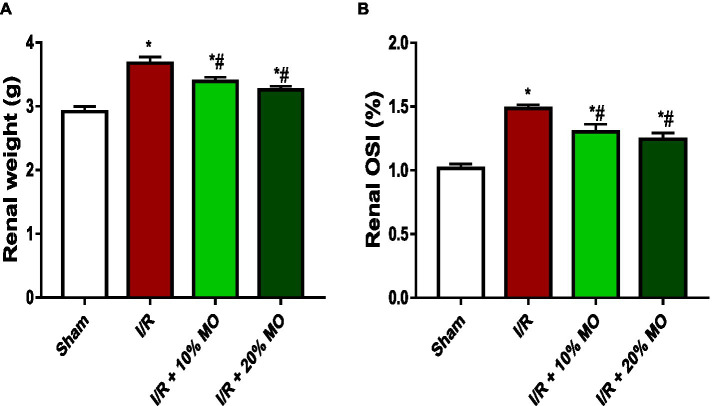
Effect of renal ischaemia/reperfusion (I/R) and *Moringa oleifera*-based feed supplement (MO) on renal weight **(A)** and renal organosomatic index, OSI **(B)**. OSI: Organosomatic index, I/R: ischemia/reperfusion, MO: Moringa oleifera-based feed supplement. *p < 0.05 versus sham, #*p* < 0.05 versus I/R. Values represent the mean for six replicates ± the standard deviation.

Absolute renal weight was significantly higher in I/R rats compared to the sham-operated rats, which was inhibited by MO-fortified diet (Figure 1A). Renal OSI was significantly higher in I/R rats compared to the sham-operated rats (*p* < 0.0001), and this was attenuated by MO-based supplement (*p* = 0.0071 vs. I/R + 10%MO, *p* = 0.0007 vs. I/R + 20%MO) (Figure 1B). The effect of MO-based supplement on I/R-induced alterations in renal weight and OSI was not dose-dependent.

### Effect of MO-based feed supplement on renal histoarchitecture in I/R rats

**Figure 2:**
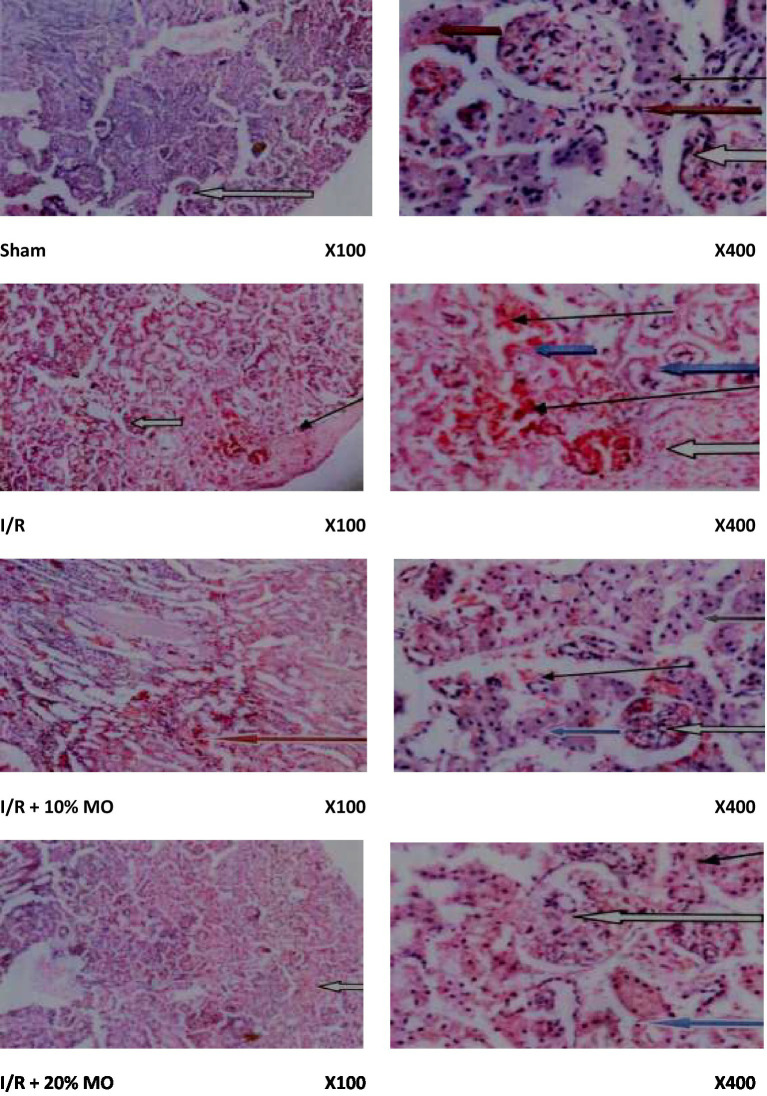
Effect of renal ischaemia/reperfusion (I/R) and *Moringa oleifera*-based feed supplement (MO) on renal histoarchitecture. The sham-operated rats showed preserved renal cortex with normal glomeruli, mesengial cells, and capsular spaces (white arrow). The interstitial spaces appeared normal (slender arrow). The rats in the I/R group showed poor renal histoarchitecture. The glomerular capillaries appeared congested (white arrow), the renal tubules showed desquamation with some obvious tubular necrosis (blue arrow), and the interstitial spaces showed moderate hemorrhage and vascular congestion (slender arrow). The rats in the I/R + 10% MO group showed congested glomerular capillaries (white arrow), desquamated renal tubules with reduced laminar space (blue arrow), and mild hemorrhage in the interstitial space (slender arrow). The medullary ray showed moderate hemorrhage within the inter-tubular spaces (red arrow). The rats in the I/R + 20% MO group showed congested glomerular capillaries (white arrow), desquamated renal tubules with reduced laminar space (blue arrow), normal cortical interstitial space (slender arrow), and hemorrhagic interstitial spaces within the collecting tubules (black arrow).

Findings of the histopathological examinations are as shown in Figure 2. The sham-operated rats showed preserved renal cortex with normal glomeruli, mesengial cells, and capsular spaces. The interstitial spaces appeared normal. The rats in the I/R group showed poor renal histoarchitecture. The glomerular capillaries appeared congested, the renal tubules showed desquamation with some obvious tubular necrosis, and the interstitial spaces showed moderate hemorrhage and vascular congestion. The rats in the I/R + 10% MO group showed congested glomerular capillaries, desquamated renal tubules with reduced laminar space, and mild hemorrhage in the interstitial space. The medullary ray showed moderate hemorrhage within the inter-tubular spaces. The rats in the I/R + 20% MO group showed congested glomerular capillaries, desquamated renal tubules with reduced laminar space, normal cortical interstitial space, and hemorrhagic interstitial spaces within the collecting tubules.

### Effect of MO-based feed supplement on renal function in I/R rats

**Figure 3:**
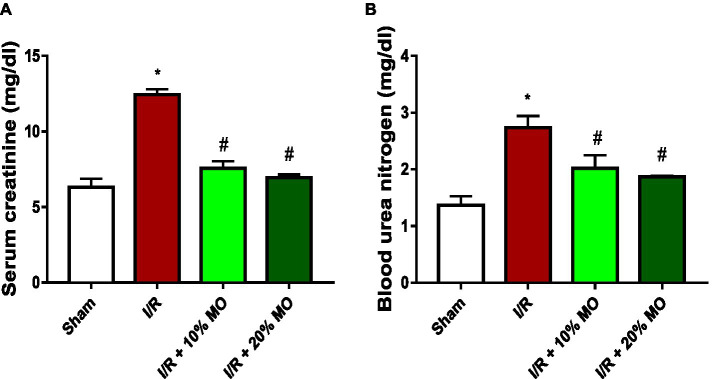
Effect of renal ischaemia/reperfusion (I/R) and *Moringa oleifera*-based feed supplement (MO) on renal function indices; serum creatinine **(A)** and blood urea nitrogen **(B)**. I/R: ischemia/reperfusion, MO: *Moringa oleifera*-based feed supplement. *p < 0.05 versus sham, #*p* < 0.05 versus I/R. Values represent the mean for six replicates ± the standard deviation.

Serum creatinine was significantly raised in the I/R animals compared with the sham-operated rats (*p* = 0.0005). MO-based feed supplement led to remarkable reduction in serum creatinine in I/R rats (*p* = 0.0012 vs. I/R + 10%MO, *p* = 0.0007 vs. I/R + 20%MO). The effect of MO-based supplement on I/R-induced rise in serum creatinine was not dose-dependent (Figure 3A). Similarly, BUN was significantly elevated in the I/R animals compared with the sham-operated rats (*p* = 0.0051), which was markedly inhibited by MO-based supplement in a dose-independent manner (*p* = 0.0489 vs. I/R + 10%MO, *p* = 0.0260 vs. I/R + 20%MO) (Figure 3B).

### Effect of MO-based feed supplement on renal oxidative stress markers and antioxidants in I/R rats

**Figure 4:**
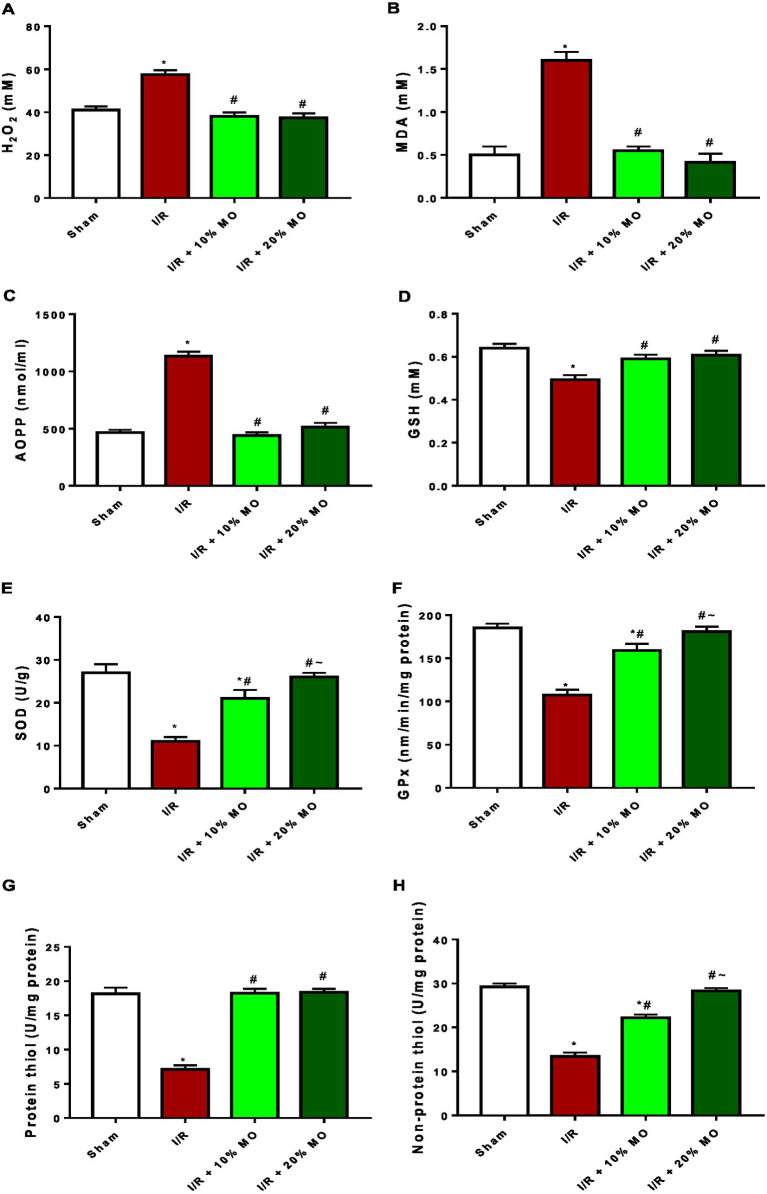
Effect of renal ischaemia/reperfusion (I/R) and *Moringa oleifera*-based feed supplement (MO) on renal oxidative stress markers and antioxidants; H_2_O_2_ **(A)**, MDA **(B)**, AOPP **(C)**, GSH **(D)**, SOD **(E)**, GPx **(F)**, protein thiol **(G)**, and non-protein thiol **(H)**. I/R: ischemia/reperfusion, MO: *Moringa oleifera*-based feed supplement, H_2_O_2_: hydrogen peroxide, MDA: malondialdehyde, AOPP: advanced oxidized protein product, GSH: reduced glutathione, SOD: superoxide dismutase, GPx: glutathione peroxidase. *p < 0.05 versus sham, #*p* < 0.05 versus I/R, ~*p* < 0.05 versus I/R + 10% MO. Values represent the mean for six replicates ± the standard deviation.

The levels of hydrogen peroxide radical was significantly increased in the kidney of I/R animals compared with the sham-operated rats (*p* < 0.0001). Ingestion of MO-based feed supplement abrogated I/R-induced rise in renal hydrogen peroxide concentration (*p* < 0.0001 vs. I/R + 10%MO, *p* < 0.0001 vs. I/R + 20%MO) (Figure 4A). Also, the concentrations of MDA (*p* < 0.0001) and AOPP (*p* < 0.0001) were significantly elevated in I/R rats compared with the sham-operated animals. Ingestion of MO-based feed supplement for 14 days prior to I/R markedly prevented I/R-induced rise in MDA (*p* < 0.0001 vs. I/R + 10%MO, *p* < 0.0001 vs. I/R + 20%MO) and AOPP levels (*p* < 0.0001 vs. I/R + 10%MO, *p* < 0.0001 vs. I/R + 20%MO) in the kidney (Figures 4B,C). The effect of MO-based supplement on I/R-induced rise in renal concentrations of hydrogen peroxide radical, MDA, and AOPP was not dose-dependent (Figures 4A–C). GSH (*p* = 0.001) concentration and SOD (*p* < 0.001) and GPx activities (*p* < 0.001) in the kidney were considerably reduced in I/R rats compared with the sham-operated rats. Ingestion of MO-based feed suplement alleviated I/R-led reduction in renal GSH concentration (*p* = 0.0018 vs. I/R + 10%MO, *p* = 0.0006 vs. I/R + 20%MO) and SOD (*p* = 0.0003 vs. I/R + 10%MO, *p* < 0.0001 vs. I/R + 20%MO) and GPx activities (*p* < 0.0001 vs. I/R + 10%MO, *p* < 0.0001 vs. I/R + 20%MO) (Figures 4D–F). Although the effect of MO-based supplement ingestion on GSH was not dose-dependent, it was dose-dependent on renal SOD and GPx activities in I/R rats. The levels of protein thiol (*p* < 0.0001 vs. I/R + 10%MO, *p* < 0.0001 vs. I/R + 20%MO) and non-protein thiol (*p* < 0.0001 vs. I/R + 10%MO, *p* < 0.0001 vs. I/R + 20%MO) were significantly reduced in the kidney of I/R rats compared with the sham-operated rats. I/R-driven reduction in protein thiol (*p* < 0.0001 vs. I/R + 10%MO, *p* < 0.0001 vs. I/R + 20%MO) and non-protein thiol (*p* < 0.0001 vs. I/R + 10%MO, *p* < 0.0001 vs. I/R + 20%MO) was inhibited by ingestion of MO-based feed suplement. Although the effect of ingesting MO-based supplement on protein thiol was not dose-dependent, it was dose-dependent on non-protein thiol in I/R rats (Figures 4G,H).

### Effect of MO-based feed supplement on renal inflammatory markers in I/R rats

**Figure 5:**
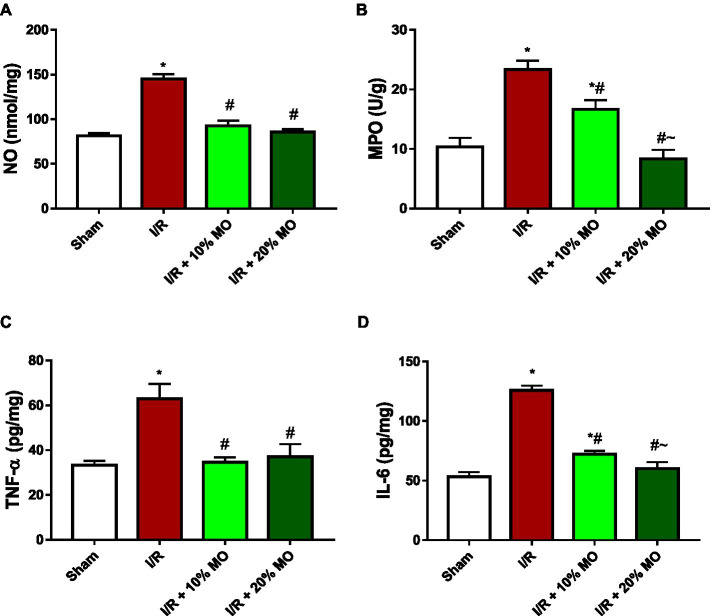
Effect of renal ischaemia/reperfusion (I/R) and *Moringa oleifera*-based feed supplement (MO) on renal inflammatory markers; NO **(A)**, MPO **(B)**, TNF-α **(C)**, and IL-6 **(D)**. I/R: ischemia/reperfusion, MO: *Moringa oleifera*-based feed supplement, NO: nitric oxide, MPO: myeloperoxidase, TNF-α: tumour necrosis factor-α, IL-6: interleukin-6. *p < 0.05 versus sham, #*p* < 0.05 versus I/R, ~*p* < 0.05 versus I/R + 10% MO. Values represent the mean for six replicates ± the standard deviation.

The level of NO in the kidney was significantly elevated in I/R rats (*p* < 0.001). The observed I/R-induced rise in renal NO was prevented by MO-based feed supplement (*p* < 0.0001 vs. I/R + 10%MO, *p* < 0.0001 vs. I/R + 20%MO) (Figure 5A). The activity of MPO in the kidney of I/R rats was significantly increased compared to the sham-operated rats (*p* < 0.001), and this rise was dose-dependently attenuated by MO-based feed supplement (*p* = 0.0031 vs. I/R + 10%MO, *p* < 0.0001 vs. I/R + 20%MO) (Figure 5B). The concentrations of TNF-α (*p* < 0.001) and IL-6 (*p* < 0.001) were increased in the kidney of I/R rats compared with the sham-operated animals. Although MO-based feed supplement inhibited I/R-induced TNF-α rise in a dose-independent manner (Figure 5C) (*p* < 0.0001 vs. I/R + 10%MO, *p* < 0.0001 vs. I/R + 20%MO), it prevented I/R-induced IL-6 rise in a dose-dependent manner (*p* < 0.0001 vs. I/R + 10%MO, *p* < 0.0001 vs. I/R + 20%MO) (Figure 5D).

### Effect of MO-based feed supplement on renal apoptosis markers in I/R rats

**Figure 6:**
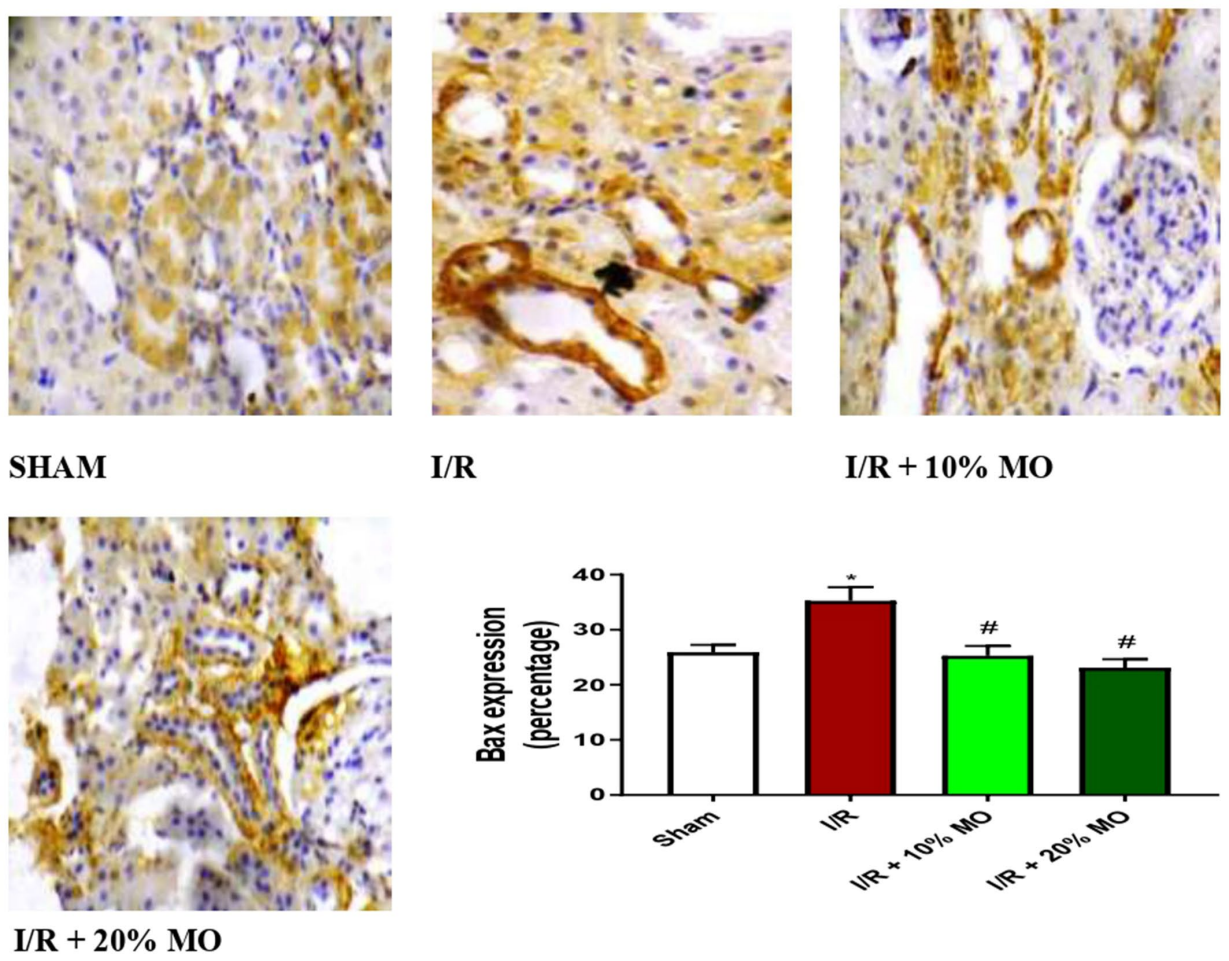
Effect of renal ischaemia/reperfusion (I/R) and *Moringa oleifera*-based feed supplement (MO) on renal Bax expression. I/R, ischemia/reperfusion; MO, *Moringa oleifera*-based feed supplement; Bax, Bcl-2-associated X protein. ^*^p < 0.05 versus sham, #*p* < 0.05 versus I/R. Values represent the mean for six replicates ± the standard deviation.

**Figure 7:**
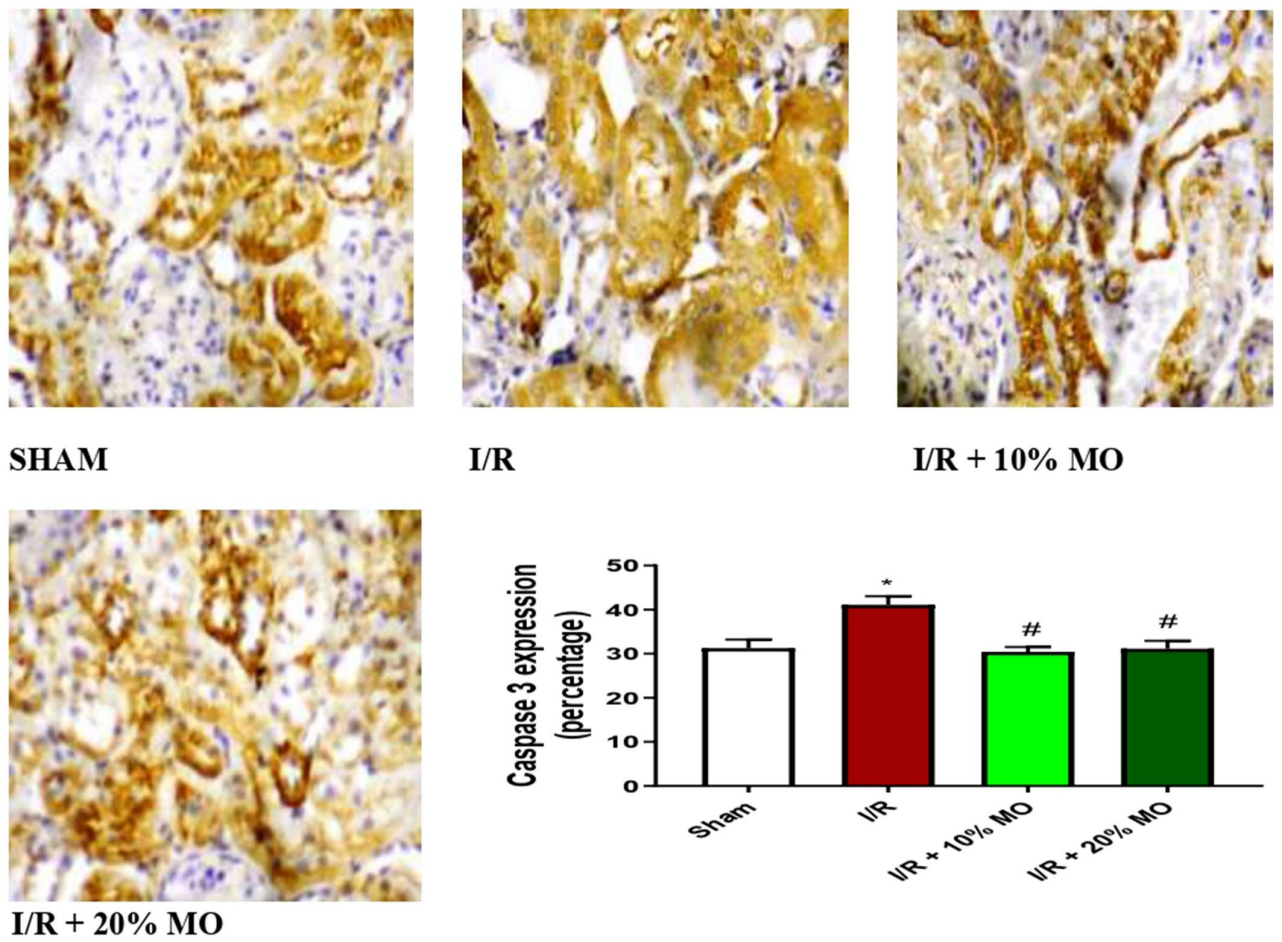
Effect of renal ischaemia/reperfusion (I/R) and *Moringa oleifera*-based feed supplement (MO) on renal caspase 3 expression. I/R, ischemia/reperfusion; MO, *Moringa oleifera*-based feed supplement, caspase 3: cysteine-aspartic protease 3. ^*^p < 0.05 versus sham, # *p* < 0.05 versus I/R. Values represent the mean for six replicates ± the standard deviation.

The expression of Bax protein was upregulated in the kidney of I/R rats compared with the sham-operated rats (*p* = 0.024), and this rise was inhibited by MO-based feed supplement (*p* = 0.0189 vs. I/R + 10%MO, *p* = 0.0093 vs. I/R + 20%MO) (Figure 6). Besides, I/R led to upregulation of caspase 3 in the kidney of I/R rats compared with the sham-operated rats (*p* = 0.0162). The rise in caspase 3 expression in I/R rats was prevented by MO-based feed supplement (*p* = 0.0122 vs. I/R + 10%MO, *p* = 0.0157 vs. I/R + 20%MO) (Figure 7). Interestingly, the downregulation of Bax and caspase 3 expression in I/R rats by MO-based feed supplement was not dose-dependent.

## Discussion

Findings of this study revealed that MO-based feed supplement significantly improved renal function in I/R rats. Our data demonstrated that MO-fortified feed inhibited I/R-induced imbalance between oxidants and antioxidants. In addition, MO-based feed supplement ameliorated the rise in inflammatory mediators such as NO, MPO activity, TNF-α, and IL-6 in the kidney of I/R rats. Furthermore, renal I/R-induced upregulation of Bax and caspase 3 in the kidney was prevented by MO-based feed supplement. These findings were coupled with MO-based feed supplement-led restoration of I/R-induced rise in absolute renal weight and renal OSI, and distortion of renal histoarchitecture.

Upregulation of inflammatory cytokines is central in the pathogenesis of renal injury in I/R (50, 51). The present finding that renal I/R led to a rise in NO content, MPO activity, and TNF-α and IL-6 levels agrees with previous reports on I/R injury (52, 53). The observed rise in renal NO may be iNOS-dependent (29), and possibly increased binding of NO to the thiol groups of glyceraldehyde-3-phosphate dehydrogenase, hence suppressing glycolytic process and leading to I/R-driven renal injury (54, 55). The excess NO produced may also react with super oxide radicals to generate peroxynitrite, which is a potent oxidant that may decompose to form hydroxyl radicals and induce renal injury through lipid peroxidation, nitrosation of tyrosine molecules, and sodium ion channel inactivation (29, 55). In addition, I/R-led rise in MPO activity, a marker of neutrophil accumulation, suggests that renal I/R promoted neutrophil activation and infiltration in the kidney of I/R rat, leading to distortion of various cellular components and cellular damage (56). It is also likely that the observed cytokine storm in renal I/R is associated with phosphorylation and degradation of IkB, which results in the release of NF-kB, leading to upregulation of TNF-α and IL-6 (57). Findings of the present study that MO-fortified feed inhibited I/R-led rise in NO content, MPO activity, and TNF-α and IL-6 levels suggest that MO-based feed supplement confers reno-protection via its anti-inflammatory activity by inhibiting the accumulation of neutrophils and pro-inflammatory cytokines. This is in agreement with the anti-inflammatory activity of MO reported by Omodanisi et al. (58) and Xu et al. (59).

Our present findings that renal I/R triggered an upregulation of Bax and caspase 3 expression confirms the findings of previous studies that demonstrated that renal I/R caused apoptosis in the kidney (60, 61) through upregulation of Bax and caspase 3 expressions (62). Caspase 3 plays a central role in apoptosis (30, 63) and may be activated by Bax (64). Based on the present observation that MO-based feed supplement suppressed Bax and caspase 3 expression, it is plausible to infer that MO-based feed supplement inhibited the activation of Bax by preventing the translocation of Bax from the cytosol into the mitochondrial outer membrane and/or its binding with BH3-only proteins (64), thus preventing Bax activation. It is also likely that MO-based feed supplement suppresses Bax oligomerization to form pores in the mitochondrial outer membrane to trigger the release of cytochrome c into the cytosol, thus preventing caspase 3 activation (64). These findings proved that MO-based feed supplement inhibited I/R-induced renal injury via a Bax/caspase 3-mediated mechanism.

Oxidative stress has been implicated in the pathogenesis of I/R-induced renal injury (5, 65). This study revealed that renal I/R induced oxidative stress in the kidney of I/R rat evidenced by the imbalance between oxidants and antioxidants. This is in agreement with previous studies that demonstrated the role of oxidative stress in renal I/R injury (65, 66). The rise in renal hydrogen peroxide radical and NO may explain the observed I/R-driven oxidative stress. Interestingly, a 14-day course of MO-based feed supplement was sufficient to curtail I/R-induced oxidative renal injury. This corroborates previous findings that MO exerts antioxidant activities (58, 59). These findings suggest that MO-based feed supplement inhibited I/R-induced inflammatory response and apoptosis in the kidney of I/R rat via inhibition of oxidative stress, possibly through downregulation of reactive oxygen species (ROS) and cytokine generation (63), suppression of neutrophil infiltration (67), and inhibition of Bax-induced caspase 3 activation (62).

Renal I/R injury is a clinical challenge with imminent AKI (4) if not promptly managed. The current finding that renal I/R induced gross and histoarchitectural distortions in the kidney was associated with impaired renal function, and agrees with previous findings (4, 68). The ability of MO-based feed supplement to maintain the gross and histoarchitectural integrity of the kidney as well as renal function is, at least partly, attributable to its antioxidant and anti-inflammatory activities. MO-based feed supplement may be a potential nutritional supplement that confers reno-protection in renal I/R injury. Furthermore, it may offer hope for preventing renal IRI and possibly IRI in other organs if and when it occurs since the MO is taken in the diet rather than in regimented doses. The absence of dose dependence may even suggest that lower degrees of supplementation than 10 percent may be equally effective in preventing IRI in real life situations.

The beneficial role of MO-based food supplement demonstrated in the present study may be attributed to its constituent bioactive compounds. According to reports, hydrazine exhibits antioxidant properties (69), while 1,3-dioxolane has been revealed to possess radical-scavenging properties (70). Also, the anti-inflammatory properties of oleic acid have been reported (71). These molecules may work in synergy to exert a potent antioxidant and anti-inflammatory activity that is sufficient to suppress intestinal I/R-induced renal injury.

In conclusion, MO-based feed supplement may prevent renal injury. MO-based feed supplement inhibited inflammatory response and apoptosis in the kidney of I/R rat by downregulating Bax/caspase 3 signaling via inhibiting oxidative stress to improve renal function, thus providing a promising and prospective natural source of bioactives for the prevention of renal I/R injury. However, other molecular mechanisms of action are required to fully elucidate the role of MO-based feed supplement in preventing I/R-induced renal injury.

## Data availability statement

The original contributions presented in the study are included in the article/supplementary material, further inquiries can be directed to the corresponding author.

## Ethics statement

The animal study was approved by ethical approval was issued by The Ministry of Health, Oyo State, Nigeria (Approval number: AD13/479/44406). The study was conducted in accordance with the local legislation and institutional requirements.

## Author contributions

OA: Project administration, Resources, Writing – review & editing, Conceptualization, Funding acquisition, Investigation, Methodology, Supervision, Validation. TA: Project administration, Resources, Writing – original draft, Writing – review & editing. SH: Investigation, Project administration, Resources, Writing – review & editing. MH: Project administration, Writing – review & editing. VE: Project administration, Writing – review & editing. RAj: Investigation, Project administration, Resources, Writing – review & editing. BA: Project administration, Resources, Writing – review & editing. RAk: Project administration, Resources, Writing – original draft, Writing – review & editing.
